# Exogenous abscisic acid and sodium nitroprusside regulate flavonoid biosynthesis and photosynthesis of *Nitraria tangutorum* Bobr in alkali stress

**DOI:** 10.3389/fpls.2023.1118984

**Published:** 2023-03-15

**Authors:** Jie Zhang, Kai Cheng, Xinyue Liu, Zhichao Dai, Lingling Zheng, Yingchun Wang

**Affiliations:** Key Laboratory of Forage and Endemic Crop Biotechnology, Ministry of Education, School of Life Sciences, Inner Mongolia University, Hohhot, China

**Keywords:** *Nitraria tangutorum* Bobr, ABA and NO, photosynthetic efficiency, flavone pathway, anthocyanin accumulation, alkali stress

## Abstract

Abscisic acid (ABA) and nitric oxide (NO) are involved in mediating abiotic stress-induced plant physiological responses. *Nitraria tangutorum* Bobr is a typical salinized desert plant growing in an arid environment. In this study, we investigated the effects of ABA and NO on *N.tangutorum* seedlings under alkaline stress. Alkali stress treatment caused cell membrane damage, increased electrolyte leakage, and induced higher production of reactive oxygen species (ROS), which caused growth inhibition and oxidative stress in *N.tangutorum* seedlings. Exogenous application of ABA (15μm) and Sodium nitroprusside (50μm) significantly increased the plant height, fresh weight, relative water content, and degree of succulency in *N.tangutorum* seedlings under alkali stress. Meanwhile, the contents of ABA and NO in plant leaves were significantly increased. ABA and SNP can promote stomatal closure, decrease the water loss rate, increase leaf surface temperature and the contents of osmotic regulator proline, soluble protein, and betaine under alkali stress. Meanwhile, SNP more significantly promoted the accumulation of chlorophyll a/b and carotenoids, increased quantum yield of photosystem II (φPSII) and electron transport rate (ETRII) than ABA, and decreased photochemical quenching (qP), which improved photosynthetic efficiency and accelerated the accumulation of soluble sugar, glucose, fructose, sucrose, starch, and total sugar. However, compared with exogenous application of SNP in the alkaline stress, ABA significantly promoted the transcription of *NtFLS*/*NtF3H*/*NtF3H*/*NtANR* genes and the accumulation of naringin, quercetin, isorhamnetin, kaempferol, and catechin in the synthesis pathway of flavonoid metabolites, and isorhamnetin content was the highest. These results indicate that both ABA and SNP can reduce the growth inhibition and physiological damage caused by alkali stress. Among them, SNP has a better effect on the improvement of photosynthetic efficiency and the regulation of carbohydrate accumulation than ABA, while ABA has a more significant effect on the regulation of flavonoid and anthocyanin secondary metabolite accumulation. Exogenous application of ABA and SNP also improved the antioxidant capacity and the ability to maintain Na^+^/K^+^ balance of *N. tangutorum* seedlings under alkali stress. These results demonstrate the beneficial effects of ABA and NO as stress hormones and signaling molecules that positively regulate the defensive response of *N. tangutorum* to alkaline stress.

## 1 Introduction

Soil salinization is an important factor restricting agricultural development (Delang, 2018). The harmful salts in salinized soil include NaCl and Na_2_SO_4_, as well as alkali salts, mainly Na_2_CO_3_ and NaHCO_3_ (Li et al., 2016). Alkali stress is highly destructive to the ecology, affecting the growth of plants, seriously affecting the soil structure, and increasing soil nitrification (Schroeder et al., 2013; Fancy et al., 2017). Currently, most studies focus on salt stress, and there are few studies on alkali stress and wild plants with strong tolerance to this environment. In general, plants produce a series of defensive responses under adverse conditions (Zhu, 2002; Zhu, 2016). Stress hormones [e.g., abscisic acid (ABA), jasmonic acid (JA), and methyl jasmonate (MeJA)] and signaling molecules [e.g., NO, Ca^2+^, and H_2_S] play important regulatory roles in this process(Huot et al., 2014; Petrov et al., 2015; Zhu, 2016; Fancy et al., 2017; Guo et al., 2018).

ABA is an important plant stress hormone. Multiple studies have shown that plants can enhance their tolerance to stress by regulating the content of ABA in their bodies and then regulating a series of physiological reactions (Gai et al., 2020). For example, it can promote stomatal closure, improve photosynthesis and antioxidant enzyme activity, and accelerate the accumulation of secondary metabolites (Wang, C et al., 2020). Tomatoes and wheat can participate in stomatal movement under drought stress through ABA, thus controlling transpiration and reducing water loss (Aroca et al., 2008; Du et al., 2013). ABA can improve the photosynthetic efficiency of plants under stress by regulating a series of photosynthetic indexes, such as chlorophyll content and photosynthetic parameters, to adapt to or tolerate the adverse effects of environmental stress (Wang, C et al., 2020). Studies have shown that exogenous application of ABA can alleviate the chlorophyll loss of plants under drought stress, maintain high maximum photochemical efficiency PSII (Fv/Fm), Fm/Fo, φPSII, qP, and net photosynthetic rate (Pn) and low non-photochemical quenching (NPQ) and alleviate the decrease of leaf Pn and intercellular CO_2_ concentration (Ci). Thus, the photosynthetic capacity of plants under adverse conditions can be improved (Huang et al., 2017; Wang et al., 2020). ABA also plays an active role in antioxidant and osmotic regulation. For example, exogenous ABA is involved in the response of poplar to salt stress by inducing the accumulation of osmotic regulators and improving the activity of antioxidant enzymes to reduce the accumulation of Na^+^ under salt stress (Li et al., 2004). Flavonoids play an important role in plant resistance to adversity, and ABA can regulate the accumulation of flavonoids’ secondary metabolites in harsh environments (Lang et al., 2021). For example, flavonols are increased in *Ligustrum vulgare* under salinity or ultraviolet (UV) radiation stress and have a significant antioxidant function in plant photoprotection (Agati et al., 2011). Gonzalez‐Villagra and Gai et al. found that ABA treatment increased drought tolerance of *Aristotelia chilensis* by promoting the accumulation of anthocyanins in leaves and flavonoids in tea trees under drought stress (González-Villagra et al., 2019; Gai et al., 2020). Naringin, like most flavonoids, has antioxidant properties(Hasanein and Fazeli, 2014). In addition, the combined transcriptional and metabolic analysis showed that exogenous ABA significantly induced the expression of genes related to sucrose synthesis and flavonoid biosynthesis in tea under drought stress, such as UDP-glycosyltransferase (UDPGase), Sucrose phosphate synthetase (SPS), Chalcone isomerase (CHI), dihydroflavonol 4-reductase (DFR), Flavonoid 3’- hydroxylase (F3’H)and Flavonol synthase (FLS), and increased the contents of sucrose, glucose, fructose and soluble sugar, thereby enhancing drought tolerance (Thalmann and Santelia, 2017). The exogenous application of ABA also promoted the high expression of Chalcone synthetase (CHS), a key enzyme in flavonoid synthesis, and the accumulation of flavonoid metabolites, improving the drought tolerance of *pigeon pea*(Yang et al., 2021).

NO is an important gas signaling molecule. Many studies have shown that NO is involved in regulating various physiological processes of plants, including germination, root formation, seedling growth, stomatal movement, maturation, leaf senescence, and biological and abiotic stress responses (Corpas and Barroso, 2013; Groß et al., 2013; Fancy et al., 2017). Many studies have proved that the exogenous application of NO donor SNP can effectively improve plant resistance (Corpas and Barroso, 2013; Fancy et al., 2017). SNP can regulate photosynthesis under stress. For example, in chrysanthemum and wheat, SNP application alleviates the decline of transpiration rate and stomatal conductance under heat and cold stress and improves tolerance (Yang et al., 2011; Ozfidan-Konakci et al., 2020). SNP can significantly induce the increase of Chl content, Pn, Ci, and stomatal conductance (Gs) in leaves under low temperature and alkali stress and reduce PSII and NPQ, thus improving the cold tolerance and alkali resistance of cucumber and ryegrass (Liu et al., 2012; Zhang et al., 2020). NO can also regulate ions, antioxidant enzymes, and osmotic regulators in plants under stress conditions. It has been reported that NO improves ion homeostasis under plant stress by reducing oxidative damage and enhancing K^+^ and Ca^2+^ ion absorption (Procházková et al., 2013). SNP can also enhance the accumulation of antioxidants and osmotic regulators to promote cold tolerance in wheat. NO can also regulate the accumulation of secondary metabolites of flavonoids under stress, thus alleviating the growth inhibition of plants (Prakash et al., 2021). For example, SNP can improve the accumulation of phenolic substances, including flavonoids, such as vinblastine, quercetin, and chlorogenic acid, in different tissues of periwinkle seedlings under salt stress, thus reducing their oxidative damage (Yang et al., 2014). Exogenous application of NO can also regulate the anabolism of flavonoids in the tea plant (*Camellia sinensis* L.) by activating the activity of phenylalaninase (PAL) (Li et al., 2019). However, most studies have focused on model plants and cultivated crops to explore the role of ABA and NO in promoting abiotic stress tolerance of plants, and few studies have addressed the process of ABA and NO tolerance to wild plants, such as *N. tangutorum*.

*N. tangutorum* belongs to the *Nitraria L*. of *Zygophyllaceae* and is endemic to China. It is an important ecological group plant in the western desert area of China. It is highly adaptable to arid and saline-alkali desert environments. As a characteristic resource plant in central and western China and a typical representative of high-resistant and high-quality forage plants in the desert steppe, *N. tangutorum* is of great research value (Wang, B et al., 2020).Previous studies by our group have shown that external application of MeJA and SNP can improve the salt stress tolerance of *N. tangutorum* seedlings by reducing oxidative stress and ionic toxicity (Gao et al., 2021; Gao et al., 2022). However, few studies have been conducted on the tolerance of *N. tangutorum* to alkaline soil environments, especially the roles of ABA and SNP in regulating the adaptability to alkaline soil environments. In this study, we evaluated the alkaline resistance of *N. tangutorum* seedlings. We measured the activity and gene expression of anti-stress-related proteases under alkali stress and comprehensively analyzed the effects of the external application of ABA and SNP on the growth and metabolic activities of *N. tangutorum* seedlings under alkali stress from the aspects of growth status, ion homeostasis, stomatal movement, photosynthetic regulation, osmotic regulation, accumulation of flavonoids, and reactive oxygen species balance. Among these, we mainly discussed the effects of exogenous ABA and SNP on growth inhibition and physiological damage relief caused by alkali stress from the perspective of maintenance of photosynthetic efficiency, accumulation of photosynthates and flavonoids, and compared the different effects of ABA and SNP. This study will contribute to a better understanding of the role of ABA/NO signaling in regulating plant physiological responses to stress.

## 2 Materials and methods

### 2.1 Plant materials and growth conditions

In July, *N. tangutorum* seeds were collected in *Alxa Left Banner, Inner Mongolia*. After the seeds are collected, they are cleaned and stored in paper bags at 4°C. The seed must first remove the outer coat, sterilize with 5% sodium hypochlorite for 15 min, and then be sown in standard Murashige and Skoog medium that has been sterilized. The seeded bottles were placed at a temperature of 25°C, 70% humidity, and a photoperiod of (Light/Dark = 16h/8h). After 21 days, consistent seedlings were transplanted into glass tubes containing sterile modified Hoagland solution and cultured for 19 days for future experiments.

### 2.2 Plant stress treatment

Forty-day-old seedlings were treated with a modified Hoagland solution containing Alkaline stress (NaHCO_3_: Na_2_CO_3_ = 9:1; 0, 10, 30, 50, 100, and 150 mM) for 12 days, and the maximum Alkaline stress concentration tolerated by *N. tangutorum* was selected. The fluid was changed every 2 days. Ten seedlings were analyzed for every treatment, repeated five times. We selected 100 mM alkaline stress as the follow-up experiment (Wang et al., 2022).

We treated 40-day-old *N. tangutorum* seedlings with different concentrations of ABA(Coolaber Biology Co., Ltd., purity 98%) (0, 10, 15, and 30 μM) and SNP(Solarbio Biology Co., Ltd., purity 98.5%) (0, 30, 50, and 70 μM) for 6 hours in darkness in 100 mM alkaline stress to determine the effects of ABA and SNP on plants and the optimal concentration for subsequent experiments. These seedlings were then transferred to a Hogland solution containing 100 mM alkaline and grown under normal photoperiod conditions. In order to eliminate the factors of light decomposition of SNP and cPTIO and the factors of bacteria contamination in indoor operation and maintain the consistency of experimental treatment, we use the light cycle of plants (Light/Dark = 16h/8h) to spray in the ultra-clean platform at 10:00 pm every night. For 12 days, treatments were given every two days. The experiment was repeated three times with thirty seedlings. The optimal ABA and SNP concentrations were respectively determined to be 15 μM and 50 μM, and were used in subsequent experiments. The same method was used to determine the concentration of ABA inhibitor fluridone (Flu) and NO scavenger 2-(4-carboxy-2-phenyl)-4,4,5,5-tetramethy limidazoline-1-oxyl-3-oxide (cPTIO) as 5 μM and 70 μM. 40 days after planting, collect samples before treatment. The stress treatment was started on the 41st day for 12 days, and the samples after treatment were collected at 10:00 pm on the 12th day. Seedling growth parameters were measured at the end of these treatments, and samples, such as leaf, shoot and root, were collected and stored at -80°C for further analysis. A ruler was used to measure plant height elongation. An electronic balance was used to determine the fresh weight of whole seedlings.

### 2.3 Physiological and biochemical index detection

After the stress is over, measure the plant height and fresh weight. Measure the relative water content (RWC). At the end of the treatment, determine the fresh weight (W0) of the leaves (0.2 g) on the middle branch of each seedling. Then, they were soaked in distilled water for 24 hours, and the saturated fresh weight was weighed and absorbed (W1). Clean the surface water with absorbent paper, dry at 105°C for 15 minutes, then dry at -80°C to a constant weight, and record (W2). Then RWC is (W0-W2)/(W1-W2)×100%. The electrolyte permeability was measured using a conductivity meter. Take the middle new leaves (3–4 pieces) of each seedling and put them in a test tube with 1 ml of distilled water at 37°C for 3 hours. Measure and record the conductivity E1. Then, for 20 minutes, boil 100 degrees Fahrenheit water; measure and record the conductivity E2. The calculation formula is E1/E2×100%. The degree of leaf succulentism was calculated based on the fresh weight of 0.1 g of leaf. According to the formula: FW/DW to get the degree of leaf succulentism.

The content of chlorophyll was extracted by the acetone extraction method, treated in the dark for 12 hours, and calculated by measuring the absorption values at 663nm, 645nm, and 445nm wavelengths, respectively. Chia = 12.72•D663 - 2.59•D645 for chlorophyll pigment concentration; Chlorophyll b pigment concentration: Chib = 22.88•D645-4.67•D663; Carotenoid pigment concentration: Car=4.7•D440-0.27 (Ca+Cb); Concentration of total chlorophyll pigment: Total = Ca + Cb. For all the parameters measured above, 3 samples of each treatment were analyzed and repeated 3 times.

The collected frozen samples are used to detect the accumulation levels of malondialdehyde (MDA), Hydrogen peroxide(H_2_O_2_) and Superoxide anion(O_2_^-^), the content of proline and soluble sugars and proteins, and the enzyme activities of SOD, APX, GR and CAT. Antioxidant content such as GSH, GSSG, The measurement was carried out according to the instructions given by Suzhou Keming. For all the parameters measured above, 3 samples of each treatment were analyzed and repeated 3 times.

### 2.4 Diaminobenzidine and nitro blue tetrazolium staining

Diaminobenzidine (DAB) and nitro blue tetrazolium(NBT) staining are as described in the Soleibao instructions, using DAB and NBT chloride staining to detect H_2_O_2_ and O_2_^-^. We took the middle taproot of each seedling for DAB staining, immersed it in 1 mg·ml^-1^ DAB solution, and kept it in the dark at room temperature for 12 h before observing the staining. For NBT staining, the taproot (3–4 pieces) of each seedling was immersed in 1 mg·ml^-1^ NBT and kept in the dark at room temperature for 8 h before observation. Ten seedlings of each treatment were analyzed for all the parameters measured above and repeated three times (Gao et al., 2022).

### 2.5 Infrared thermal imaging and water loss rate

As previously mentioned, thermal imaging is used to monitor the temperature of plant leaves under CK and different stress treatments (Nguyen et al., 2018). We first let plants grow under normal conditions and then select seedlings with the same growth momentum to simultaneously start alkali stress and other treatments. We used a thermal imaging camera to detect and record the temperature of plant leaves after 12 days of stress. We used the method described previously and made some modifications (Leung et al., 1997). Before and after the stress treatment, the water content of plant leaves was initially weighed once, and the weight was weighed every 10 min. The water loss rate is the ratio of the weight dropped to the weight of the detached blade at 0 min. Eight leaves were selected from each strain, and the experiment was repeated three times. At the same time, take the lower epidermis of leaves after 14 days of treatment, observe the stomata and take photos.

### 2.6 Chlorophyll fluorescence imaging and determination of photosynthetic index

As previously mentioned, a plant *in vivo* imaging system was used to monitor the chlorophyll content of plants under CK and different stress treatments. After 12 days of stress, the seedlings with the same growth vigor were placed into the chlorophyll fluorescence imager, and the observation records were taken at 50 s intervals under sufficient light, following the method of (Wang and Kinoshita, 2017), with minor modifications. An LI-6400 device (LI-COR Inc, Lincoln, USA) was used to measure the Pn, Ci, and transpiration rate (Tr) of leaves of *N. tangutorum* seedlings at the same leaf position in different treatment groups.

We followed the protocol described by Lu et al. (Lu et al., 2003). At room temperature (25°C) and relative humidity of 60%, the chlorophyll fluorescence parameters Fv/Fm, φPSII, qP, and ETRII were measured using a portable chlorophyll fluorescence meter (PAM-2500) of leaves in different treatment groups. Before determination, the plant must adapt to dark conditions for 30 min (we used the 3^rd^ to 6^th^ fully expanded leaf from the top of the plant).

### 2.7 Sugar and starch content measurement

The sugar substances and starch were determined according to the instructions of the Suzhou Keming Reagent Kit. Take 0.1g of tissue and add 1 ml of distilled water to grind it into homogenate, and take a water bath at 95°C for 10 minutes; 8000 g, centrifuged at 25°C for 10 minutes, and 20 ul of the supernatant are taken to be tested. Reagent II and Reagent III are mixed in 1:1 equal volume, mixed with the supernatant, and kept at 25°C for 15 minutes. The absorbance value was measured at 505 nm with the enzyme marker. The absorbance values of a blank tube, a standard tube, and a measuring tube are recorded as A1, A2, and A3, respectively. Calculate the content according to the formula: glucose content=0.5 × (A3-A1)÷(A2-A1)÷W. Similarly, the extraction and determination of fructose, sucrose, total sugar, starch, and soluble sugar were carried out step by step according to the instructions of the Suzhou Keming Reagent Kit. According to different formulas, the sequence is: fructose content = (A3-A1) ÷ (A2-A1) ÷ W; Sucrose=(A3-A1) ÷ (A2-A1) ÷ W; soluble sugar=2.34 × [(A3-A1)+0.07] ÷W; Starch content=0.289 × (A3+0.0295) ÷W; Total sugar=33.311 × [(A3-A1)+0.0507] ÷W × Dilution ratio.

### 2.8 Determination of flavonoids by LC-MS

To determine total flavonoids and flavonol, we diluted rutin standard solution in gradient, added 0.5 ml 5% NaNO_2_, mixed, and placed at room temperature for 6 min. Next, we added 0.5 ml of 10% Al (NO_3_) _2_ solution, mixed well, and left for 6 min. We then added 4 ml of 4% NaOH solution and 60% ethanol to a constant volume of 10 ml. A microplate reader measured the absorbance at 510 nm (standard curve: y = 0.0059x^+^0.0406, R^2^ = 0.9994). One gram of fresh *N. tangutorum* seedlings was added to liquid nitrogen and ground into a powder with a mortar. Ethanol (60%) was then refluxed and extracted three times. The light absorption value was determined according to the above steps (OD = 510 nm). The content of total flavonoids was calculated according to the standard curve. One gram of sample powder was added into a 100 ml conical flask with 50 ml of methanol and extracted by ultrasonic wave (500W, 40kHz) three times, 30 min each time. The filtrates were then filtered, combined, and extracted with petroleum ether at a ratio of 1:1 three times, concentrated by reducing pressure, then adding methanol to dissolve it by ultrasonic wave. Finally, this was diluted to 25 ml in a brown volumetric flask and mixed to obtain the test solution. We then determined the light absorption value at OD = 440 nm and calculated the total flavonol content relative to a standard.

Anthocyanin and proanthocyanidin content measurement: we first accurately weighed 2 mg of cyanidin-3-O- glucoside and dissolved this in 1 ml of extract (methanol:HCl:H_2_O = 80:5:15, v:v:v), which was diluted with the extract in equal gradient. Of this, 200 μl was used to determine the OD value at 520 nm and generate a standard curve (y = 0.00306x^+^0.00042, R^2^ = 0.9999). One gram of fresh sample was added into 3 ml of extraction solution. Ten minutes later, draw 200 µl of this was added to the enzyme standard plate, and the OD value was measured at 520 nm. We calculated the anthocyanin content according to the standard curve. The sample was treated with the same method, adding 385 µl methanol and 192 µl DMACA reagent [2% (w/v) DMACA was dissolved in methanol, and 6 M concentrated hydrochloric acid (1:1) (v/v)]. The absorbance value was detected at A643 by microplate, and proanthocyanidins were calculated.

The quantification of Catechin;Quercetin; Isoamnetinrh; Kaempferol; Naringenin using an HPLC–MS/MS platform as described Plant materials (50 mg FW) were frozen in liquid nitrogen, ground into powder, and extracted with 0.5 mL methanol, water, or formic acid (15:4:1, V/V/V) at 4 °C. The extract was vortexed for 10 minutes before being centrifuged at 14,000 rpm for 5 minutes at 4°C. The supernatant was collected, and the extraction steps were repeated. The combined extracts were evaporated to dryness under a stream of nitrogen gas, reconstituted in 80% methanol (V/V), ultraphoniced (1 min), and redissolved in 10 µL of methanol and passed through a 0.22 µm filter before HPLC–MS/MS analysis. The sample extracts were analyzed with an UHPLC (Agilent 1290 Infinity, Santa Clara, CA)-triple quadropole (Agilent 6430). The analytical conditions were as following, HPLC: The analysis column was an Agilent ZORBAX Eclipse Plus rapid resolution high definition C18 (octadecyl silane) column (2.1 × 50 mm, 1.8 µm); solvent system, water (0.1% aqueous formic acid), methanol; and Needle (ND) injection volume: 2 µL. as follows: (0 to 4 min, 40 to 40%; 4 to 7.5 min, 40 to 45%; 7.5 to 8 min, 45 to 48%; 8 to 9 min, 48 to 100%; 9 to 11 min, 100%). ESI-MS in negative ionization mode was used for detection with capil-lary 3 kV, gas temperature at 325°C, gas flow at 12 L/min, and nebulizer at 40 psi in multiple-reaction-monitory (MRM) mode. Chromatographic peaks were identified and con-firmed by comparison with standards of their mass-charge ratio (m/z) values, product ions, and retention times. Standard curves consisting of 3.1, 12.2, 48.8, 195.3, 781.3, 3125.0, 12500.0, 50000.0, and 200000.0 ng/mL of catechin, quercetin, Isoamnetin rhcontent; Kaempferol; Naringeninusing were used to quantify each compound. Ten fully expanded leaves from 10 independent plants subjected to each of the stress treatments and the control were pooled as one biological replicate for flavonoids quantification, and three biological replicates were performed.

### 2.9 Na^+^/K^+^ content detection

The plant leaves and roots were washed with clean water and dried to a constant weight, and 0.1 g was used for subsequent experiments. The sample was nitrated with 10% (v/v) HNO_3_ for 8 h and centrifuged at 12000 rpm for 1 min. The supernatant was analyzed using an Optima 8000 ICP-OES DV spectrometer (PerkinElmer, Inc.) following the manufacturer’s instructions. Three replicates were measured for each group and repeated three times.

### 2.10 RT-PCR and qRT-PCR

TransStart Tip Green qPCR SuperMix and Rotor-Gene Q 5plex HRM Priority Package were used for real-time PCR and melting curve analysis. The *NtACTIN* genes were used as internal controls. The primers are listed in **Table S1**. Different cDNAs were prepared from three independent RNA samples and repeated three times. The 2^-ΔΔCT^ method was used to determine the relative expression level of the target gene. For each sample, three biological replicates and three technical replicates were measured. The sequence of each gene was obtained from our previous *N. tangutorum* transcriptome data (accession no. PRJNA273347).

### 2.11 ABA and NO content measurement

Fresh leaves (1 g) were taken and crushed, and an extract solution containing 3 ml 50 mM cool acetic acid buffer (pH 3.6) and 4% zinc diacetate was added. After centrifugation at 1000 prm at 4°C for 10 min, the supernatant was taken and added with Griess reagent (1% sulfameic acid, 1% N-1-naphthalene ethylenediamine dihydrochloride, 5% phosphoric acid),incubated at room temperature for 30 min. The absorbance was determined at 540 nm for the determination of NO.

ABA was quantified on frozen leaves (1 g). The extraction and fractionation processes have previously been described (Pan et al., 2010). The phytohormones were detected *via* ultra-high performance liquid chromatography (Agilent) and triple quadrupole mass spectrometry (Agilent) using an SPE column (Strata X-C 33 mm,30 mg/ml^−1^, Phenomenex). All experiments were repeated three times.

### 2.12 Data analysis

All experimental data are expressed as mean ± standard deviation (n = 3–5). Statistical differences were evaluated by Duncan’s multiple range test using one-way analysis of variance (ANOVA). A significant difference is guaranteed at *P*<0.05. All analyses were performed using SPSS 18.0 (IBM, Armonk, NY, USA). All data statistics are graphed with Gragh8.

## 3 Results

### 3.1 ABA and SNP alleviated the growth inhibition of *N. tangutorum* seedlings under alkali stress

**Figure 1:**
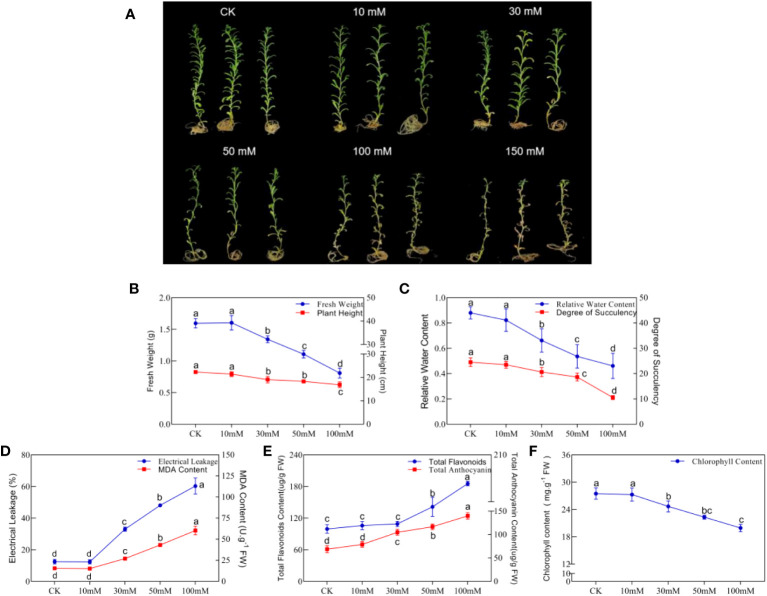
Phenotypes and indicators of *N. tangutorum* treated with different concentrations of alkaline salt(AS). **(A)**: Phenotype under different alkali treatments(AS: NaHCO_3_: Na_2_CO_3 =_ 9:1, pH=9.5); **(B)**: Fresh weight and plant height; **(C)**: Relative water content and degree of succulent; **(D)**: Eletrical leakage and MDAcontent; **(E)**: Total flavonoids and total anthocyanin content; **(F)**: Chlorophyll content. Selected 40-day-old plants were treated with different concentrations of alkaline salt(AS)(10/30/50/100/150 mM). Plants grew in Hoagland solution served as controls(CK). Each group was treated with 25 plants and repeated 3 times. Data presented are the mean ± SDs (n=3). Different letters next to the number indicate significant difference (Duncan multiple range test; *P*<0.05).

To study the effects of an alkaline salt soil on the growth and development of *N. tangutorum* seedlings, NaHCO_3_: Na_2_CO_3_ = 9:1 was used to simulate alkali stress, and related phenotypes and physiological indicators of plants were detected. We found that a low concentration of 10 mM alkali stress (NaHCO_3_: Na_2_CO_3_ = 9:1, pH = 9.5) did not affect the normal growth of plants. But 30 mM alkali stress and above significantly inhibited the growth of *N. tangutorum* plants, and the inhibition increased with the increase in concentration (**Figure 1A**). The main manifestations were decreased plant height, fresh weight (FW), relative water content (RWC) and chlorophyll (ChI) (**Figures 1B, C, F**), and increased electrolyte leakage (EL) and MDA content (**Figure 1D**). Under 100 mM alkali stress, plant damage was serious; plant height, FW, RWC, succulent degree (DS), and ChI were significantly decreased compared with CK. FW and DS were significantly decreased by two times, and MDA and EL significantly increased by two times and three times, respectively. It should be noted that the contents of total flavonoids and total anthocyanins in plants were gradually increased in a concentration-dependent manner under alkali stress of 30 mM and above, and reached 1.86 times and 2.05 times, respectively, of CK at 100 mM alkali stress (**Figure 1E**). Because 150 mM alkali stress caused the death of the plants, the correlation values were not shown. The above studies show that alkali stress severely inhibited the normal growth of *N. tangutorum* seedlings, and 100 mM alikali stress (NaHCO_3_: Na_2_CO_3_ = 9:1) was the most significant, which was used for the subsequent experiments.

Many reports have shown that exogenous ABA and SNP can improve plant adaptability to abiotic stress. To investigate the effect of exogenous ABA and SNP on alleviating the adverse effects of alkali stress on the growth of *N. tangutorum* seedlings, different concentrations of ABA (10, 15, and 30 μM) and SNP (30, 50, and 70 μM) were combined based on 100 mM alkali stress. Seedling growth and associated physiological indicators were measured (**Figure S1** and **Table 1**). The results showed that low concentrations of ABA (10 μM) and SNP (30 μM) had some alleviating effects on RWC and DS of seedlings under alkali stress but had little effect on plant height and FW. Exogenous application of 15 μM ABA and 50 μM SNP significantly improved plant growth inhibition. Compared with the single alkali stress, the RWC and DS respectively increased by more than 30% and 40%, and the FW increased by 50% and 20%. At the same time, exogenous ABA treatment increased plant height by 25%, but SNP treatment had no significant change. Compared with 50 μM SNP treatment, 15 μM ABA had more significant effects on physiological indexes. When ABA and SNP concentrations reached 30 μM and 70 μM, the growth state and physiological indexes of seedlings were not significantly different from those of 15 μM ABA and 50 μM SNP, respectively. Exogenous application of No-specific scavher cPTIO (70 μM) or ABA synthesis inhibitor Fluorine (5 μM) reversed the effects of ABA and SNP on plant growth under alkali stress. Therefore, 15 μM ABA and 50 μM SNP were considered to be the optimal treatment concentrations for subsequent experiments (**Figure S1** and **Table 1**).

**Table 1:** Physiological indicators of *N. tangutorum* applied various concentrations of ABA and SNP under 100mM alkali stress.

Physiological Indicators	Fresh Weight (g)	Plant Height (cm)	Relative Water Content (%)	Degree of Succulency (FW/DW)
CK	1.56 ± 0.12a	25.3 ± 0.02a	93 ± 0.01a	26.9 ± 0.14a
100 mM aline-alkali(AS)	0.85 ± 0.08c	18.3 ± 0.04c	39 ± 0.04d	9.7 ± 0.13d
AS+10 μM ABA	0.91 ± 0.08c	18.2 ± 0.03c	47 ± 0.02c	11.4 ± 0.04c
AS+15 μM ABA	1.24 ± 0.03b	22.8 ± 0.03b	51 ± 0.04b	16.1 ± 0.08b
AS+30 μM ABA	1.13 ± 0.01b	23.5 ± 0.09b	53 ± 0.05b	16.5 ± 0.015b
AS+30 μM SNP	0.96 ± 0.05c	18.1 ± 0.07c	45 ± 0.06d	11.9 ± 0.026c
AS+50 μM SNP	1.06 ± 0.08b	18.7 ± 0.05c	52 ± 0.13c	13.5 ± 0.14c
AS+70 μM SNP	1.05 ± 0.04b	19.5 ± 0.04c	59 ± 0.09c	14.6 ± 0.02c
AS+70 μM cPTIO	0.65 ± 0.02d	17.5 ± 0.05d	25 ± 0.08e	8.3 ± 0.05e
AS+5 μM Fluridone	0.58 ± 0.01de	17.2 ± 0.01d	14 ± 0.16f	4.2 ± 0.12f

### 3.2 Exogenous ABA and SNP further increased the content of endogenous ABA/NO and the expression levels of related genes in *N. tangutorum* seedlings under alkali stress

To further evaluate the effects of alkali stress and combination treatment on ABA and NO levels and signaling pathways in *N. tangutorum* seedlings, the content of ABA and NO and the expression levels of related genes were detected. We found that compared with CK, the endogenous ABA content of *N. tangutorum* seedlings was significantly increased by 5.84 times under single alkali stress and further increased to 8.21 times that of the CK group under combined ABA (**Figure S2C**), but there was no significant difference in ABA content under combined SNP or single alkali stress. The expression of related genes also showed the same trend. Alkali stress alone or combined application of ABA and SNP could significantly increase the NO content in plants, which was 1.87 times, 4.28 times, and 3.41 times that in the CK group, respectively (**Figure S2B**). The application of fluorine and cPTIO has the opposite effect. Real-time fluorescence quantitative PCR was used to detect the expression levels of genes related to NO and ABA biosynthesis and key elements of ABA signaling pathways. The results showed that they participated in the biosynthesis of NO (*NtNOA1*, *NtNR2*) and ABA (*NtNCED1/3/4/5*, *NtAAO*, *NtSDR*), and the expression levels of genes related to key elements of ABA signaling pathway (*NtPYL2/6*, *NtPP2C*, *NtABF1/3*, *NtSnRK2.2/2.3*) were significantly induced under single alkali stress (**Figure S2A**). The combined application of ABA and SNP under alkali stress further induced the expression of these genes to varying degrees, and the effect of ABA was more obvious than SNP. These results indicate that alkali stress induced the accumulation of endogenous NO and ABA in plants, and the combination of exogenous NO and ABA could further enhance the accumulation of ABA and SNP in plants and alleviate the growth inhibition of plants under alkali stress. It was speculated that ABA and NO might be involved in regulating the response of *N. tangutorum* seedlings to alkali stress.

### 3.3 ABA and SNP enhanced the oxidative balance of *N. tangutorum* seedlings under alkali stress by improving the efficiency of the antioxidant system

**Figure 2:**
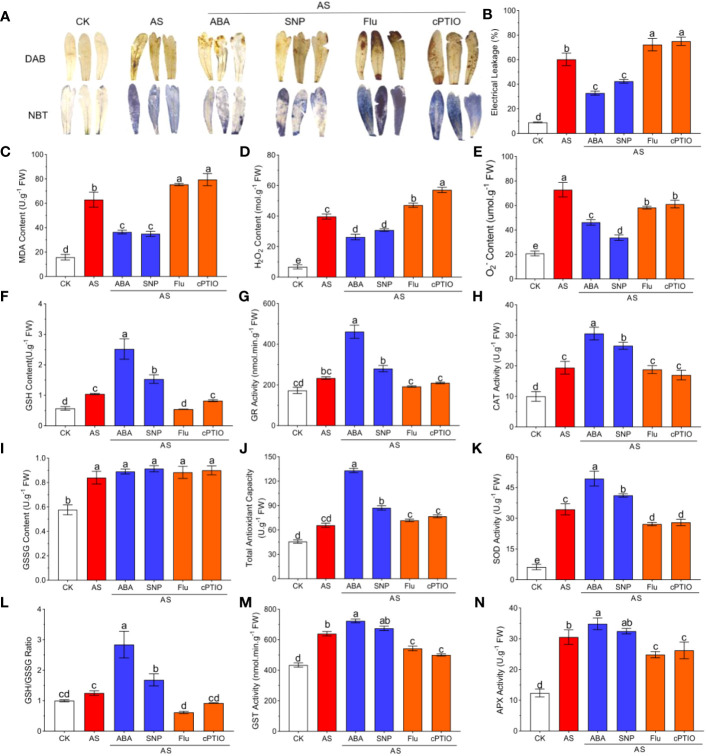
ABA and SNP alleviate ROS accumulation and accelerates antioxidant defenses under 100mM alkali stress in leaves (AS: NaHCO_3_: Na_2_CO_3 =_ 9:1, pH=9.5). Growth status of *N. tangutorum* 40-day-old seedlings treated with different treatments (100 mM alkali, 100 mM alkali +15 μm ABA/5 μm Flu, 100 mM alkali +50 μm SNP/70 μm cPTIO) for 12 days. **(A)**: DAB and NBT staining of the leaves; **(B)**: Electrical leakage (EL); **(C)**: MDA; **(D)**: Hydrogen peroxide content (H_2_O_2_); **(E)**: Superoxide anion content (O_2_^-^); **(F)**: GSH content; **(G)**: GR activity; **(H)**:CAT activity; **(I)**: GSSG content;**(J)**: Total antioxidant capacity; **(K)**: SOD activity; **(L)**: GSH/GSSG ratio; **(M)**: GST activity; **(N)**: APX activity. Bars represent the means ± SDs of three replicates. Significant differences among treatments are indicated by different letters within a panel based on Duncan’s multiple range test (*P* < 0.05).

ABA and SNP not only alleviated plant growth inhibition but also reduced oxidative damage suffered by plants. NBT and DAB staining intuitively showed the degree of oxidative damage to leaves. The accumulation of ROS in leaves induced by alkali stress significantly reduced the accumulation of blue and brown precipitates in leaves (**Figure 2A**). The content of ROS in leaves was determined by the microplate method. The results showed that alkali stress significantly aggravated MDA and EL and significantly induced the production of H_2_O_2_, and O_2_^-^, which were 3.92, 6.62, 5.93, and 3.49 times greater than for the CK group, respectively (**Figures 2B–E**). External application of 15 μM ABA and 50 μM SNP significantly decreased MDA, EL and H_2_O_2_ by 20–40% (**Figures 2B–D**). Among them, ABA and SNP had the strongest regulatory effects on O_2_^-^, with significant decreases of 35% and 49%, respectively (**Figure 2E**). The application of Fluorine and cPTIO has the opposite effect (**Figure 2**).

Plants usually turn on the oxidative defense system to resist oxidative damage caused by stress. To evaluate the effects of antioxidant enzymes and antioxidants on alleviating oxidative damage in *N. tangutorum* seedlings under alkali stress and the role of ABA and NO in regulating the antioxidant system, we examined the content of antioxidant enzymes under different treatments. Compared with untreated plants, alkali stress significantly induced the activities of GR, GST, CAT, APX, and SOD, decreased the GSSG content and the GSH/GSSH ratio, and increased the GSH content and total antioxidant capacity (**Figures 2F–N**). Compared with the seedlings treated with alkali stress alone, the activities of the above antioxidant enzymes and antioxidant content were further significantly increased by 1.21-1.62 times under the treatment of ABA and SNP (**Figures 2F–H, J–L**). The application of Fluorine and cPTIO has the opposite effect. These results suggest that exogenous application of ABA and SNP can further activate antioxidant defense in seedlings under alkali stress and reduce ROS accumulation (**Figures 2F–N**).

### 3.4 ABA and SNP promoted Na^+^/K^+^ balance in *N. tangutorum* seedlings under alkali stress

**Figure 3:**
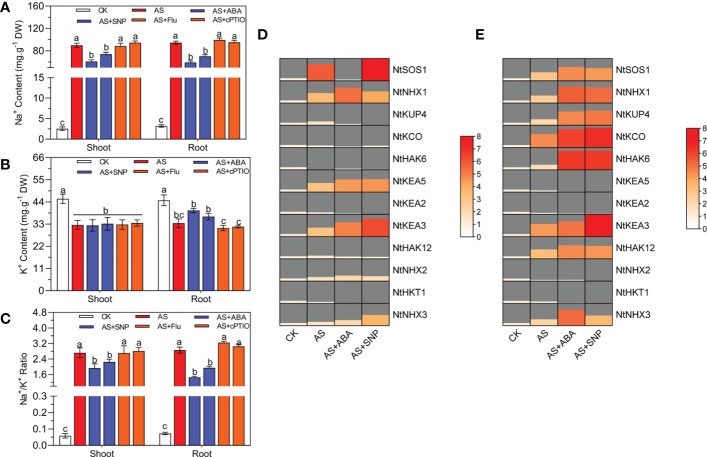
Ion content and quantification of related genes in *N. tangutorum* seedlings under different treament. 40-day-old seedlings were treated with different treatments (100 mM alkali, 100 mM alkali +15 μm ABA/5 μm Flu, 100 mM alkali +50 μm SNP/70 μm cPTIO) for 12 days(AS : NaHCO_3_: Na_2_CO_3 =_ 9:1, PH=9.5). Plants grew in Hoagland solution served as controls (CK). **(A)** Na^+^ content, **(B)** K^+^ content, **(C)** the Na^+^/K^+^ ratios, **(D, E)** The expression level of ion transporter genes in leaf and root, including *NtSOS1, NtNHX1/2/3, NtKUP4, NtKCO, NtHAK6/12, NtKEA2/3/5* and *NtHKT1*. Bars represent the means ± SDs of three replicates. Significant differences among treatments are indicated by different letters within a panel based on Duncan’s multiple range test (*P* < 0.05).

Alkali stress usually causes plants to accumulate a large amount of Na^+^, resulting in the imbalance of Na^+^/K^+^homeostasis, thus affecting the normal development of plants. In this study, we also measured the Na^+^ and K^+^contents in the shoot and roots of *N. tangutorum* seedlings under alkali stress. The results showed that after 100 mM alkali stress, the Na^+^content and Na^+^/K^+^ratio in the shoot and root of seedlings increased significantly, while the K^+^content decreased significantly, resulting in significant Na^+^ toxicity. After ABA and SNP treatment, the Na^+^content and Na^+^/K^+^ratio in the shoot and root decreased significantly, alleviating the accumulation of Na^+^ caused by alkali stress. In addition, ABA and SNP also significantly promoted the accumulation of K^+^ in roots and less excessive absorption of Na^+^ in roots; At the same time, the K^+^content of leaves remained unchanged (**Figures 3A–C**). Real-time quantitative PCR analysis showed that different treatments significantly affected the expression of ion transporter-related genes. Alkali stress significantly increased the expression of plasma membrane-localized Na^+^/H^+^ antiporter (*NtSOS1*) and vacuolar Na^+^/H^+^ antiporter (*NtNHX1/2/3*) in leaves and roots. At the same time, the K^+^/H^+^ reverse transporter 5 (*NtKEA5*) located by trans Golgi was significantly increased in leaves and significantly decreased in roots, while the transcription level of the high-affinity K^+^ transporter (*NtHKT1*) located by plasma membrane was decreased in leaves and roots (**Figures 3D, E**). Compared with alkali stress alone, ABA and SNP significantly induced the expression of *NtSOS1*, *NtNHX1/2/3*, *NtKEA3/5* in leaves, and *NtSOS1*, *NtNHX1/3*, *NtKUP4*, *NtKCO*, *NtHAK6/12* in roots. In addition, ABA and SNP also decreased the expression of *NtHKT1*, *NtHAK6/12*, *NtKCO*, and *NtKUP4* in leaves, which was consistent with the change of K^+^ content in leaves (**Figure 3**). The application of Fluorine and cPTIO has the opposite effect. The results showed that both ABA and SNP could significantly promote the accumulation of K^+^ in plant roots, reduce the Na^+^/K^+^ in aboveground and underground parts, and reduce the Na^+^ toxicity induced by alkali stress.

### 3.5 Exogenous ABA and SNP can regulate stomatal opening and reduce water loss of *N. tangutorum* seedlings under alkali stress

**Figure 4:**
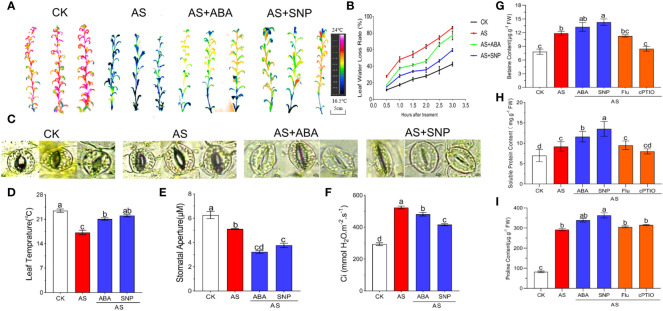
Effects of different treatments on leaf transpiration and osmomodulatory substances of *N. tangutorum* seedlings. 40-day-old seedlings were treated with different treatments (100 mM alkali, 100 mM alkali +15 μm ABA/5 μm Flu, 100 mM alkali +50 μm SNP/70 μm cPTIO) for 12 days(AS : NaHCO_3_: Na_2_CO_3 =_ 9:1, pH=9.5), and subsequent experiments are the same. Plants grew in Hoagland solution served as controls(CK). **(A, B, D)**: Infrared imaging, leaf water loss rate and leaf temperature of leaves of *N. tangutorum* seedlings under different stress treatments. **(C)** and **(E)**: stomatal phenotype and stomatal aperture of *N. tangutorum* seedlings under different treatments. **(F)**: Intercellular CO_2_ concentration; **(G)**: Betaine content; **(H)**: Soluble protein content; **(I)**: Proline content. Bars represent the means ± SDs of three replicates. Significant differences among treatments are indicated by different letters within a panel based on Duncan’s multiple range test (*P* < 0.05).

Leaf surface temperature can indicate the degree of water loss caused by transpiration. The higher the stomatal opening, the stronger transpiration, the lower the leaf surface temperature, and the more serious the water loss. Therefore, we measured the stomatal opening, leaf surface temperature, and water loss rate of *N. tangutorum* seedlings under 100 mM alkali stress with combined ABA and SNP for 12 days. The results showed that under normal growth conditions, the leaf surface temperature was about 23.1°C, and the water loss rate was the lowest. Under alkali stress, the stomatal opening of *N. tangutorum* seedlings decreased significantly, but the stress still caused an increase in water loss. The water loss rate reached the maximum within 0–3 h *in vitro*, and the leaf temperature decreased significantly to 17.2°C. After the combined application of ABA and SNP, stomata were further closed, water loss was significantly reduced compared with single alkali stress, and leaf surface temperature increased to 20.8°C and 21.1°C (**Figures 4A–E**). The contents of osmotic regulatory substances such as betaine, soluble protein and proline were significantly increased under alkali stress, indicating that *N. tangutorum* seedlings reduce water loss under stress by regulating the accumulation of osmotic substances. In addition, compared with alkali stress, the combined application of SNP induced a more significant accumulation of the above substances than ABA (**Figures 4G–I**), and the application of Fluorine and cPTIO had the opposite effect. Applying SNP can promote stomatal closure and the accumulation of osmoregulation substances of *N. tangutorum* seedlings more than ABA, thus reducing the water loss caused by alkali stress.

In addition, we also measured the intercellular CO_2_ concentration. The intercellular CO_2_ concentration increased significantly when plants were subjected to stress, but the exogenous application of ABA and SNP could significantly reduce the intercellular CO_2_ concentration, and the effect of SNP was also significantly higher than that of ABA. These results indicate that alkali stress can reduce the utilization of CO_2_ by plants, and the external application of ABA and SNP can promote the use of CO_2_ by *N. tangutorum* seedlings under alkali stress (**Figure 4F**).

### 3.6 ABA and SNP increased photosynthetic efficiency and accumulation of photosynthates in *N. tangutorum* seedlings under alkali stress

**Figure 5:**
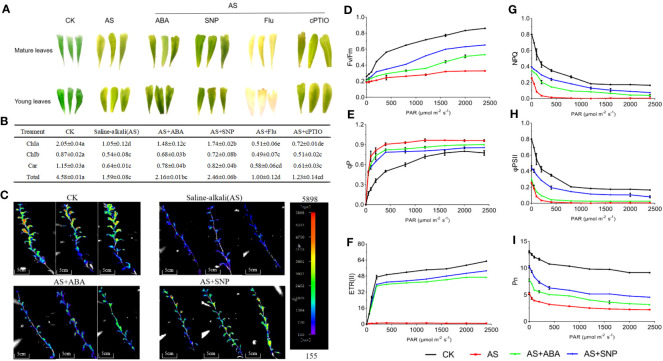
Effects of different treatments on chlorophyll content and photosynthesis in leaves of *N. tangutorum* seedlings. 40-day-old seedlings were treated with different treatments (100 mM alkali, 100 mM alkali +15 μm ABA/5 μm Flu, 100 mM alkali +50 μm SNP/70 μm cPTIO) for 12 days (AS : NaHCO_3_: Na_2_CO_3 =_ 9:1, pH=9.5), and subsequent experiments are the same. Plants grew in Hoagland solution served as controls(CK). **(A)**: Phenotypes of old and new leaves under different stress treatments; **(B)**: determination of chlorophyll content; **(C)**: Fluorescence imaging of plant chlorophyll *in vivo*; **(D–I)**: Determination of photosynthetic indexes under different treatments, including: Photochemical efficiency of photosystem II (Fv/Fm); photochemical quenching (qP); photosystem II apparent photosynthetic electron transfer efficiency (ETRII); non photochemical quenching (NPQ); quantum yield of photosystem II (φPSII); net photosynthetic rate(Pn). Bars represent the means ± SDs of three replicates. Significant differences among treatments are indicated by different letters within a panel based on Duncan’s multiple range test (*P* < 0.05).

Plants use photosynthesis to ensure an adequate energy supply to cope with adverse environmental damage. In this study, we not only examined the CO_2_ utilization efficiency of plants but also examined the changes in chlorophyll content and paid attention to a series of photosynthetic-related indicators and the accumulation of photosynthates to evaluate the damage of alkali stress on the photosynthesis of *N. tangutorum* seedlings, and the positive effect of ABA and SNP on alleviating this photosynthetic damage. In this study, the yellowing phenotype occurred in the mature and young engagement of *N. tangutorum* seedlings under alkali stress, and the contents of chlorophyll a, chlorophyll b, carotene and total chlorophyll decreased significantly. After the combined application of ABA and SNP, the yellowing of leaves was significantly alleviated, and chlorophyll content was significantly increased (**Figures 5A, B**). Chlorophyll fluorescence also showed a similar pattern. Compared with single alkali stress, leaf chlorophyll fluorescence partially recovered after ABA and SNP application. Combined SNP application had a higher fluorescence recovery and a more obvious effect than ABA (**Figure 5C**). The application of Fluorine and cPTIO has the opposite effect. This indicated that SNP could significantly reduce chlorophyll loss and reduce plant senescence than ABA. Alkali stress not only significantly inhibited the synthesis of chlorophyll in plants but also significantly reduced the Fv/Fm, ETRII, PSII, and Pn, and increased qP, thus reducing the photosynthetic efficiency of plants.Importantly, the reduction of the above indexes could be alleviated after the combination of ABA and SNP. Compared with ABA, external SNP application significantly improved Fv/Fm, ETRII, Pn and PSII and NPQ, thus more effectively alleviating the photosynthetic inhibition caused by alkali stress (**Figures 5D–I**). The application of Fluorine and cPTIO has the opposite effect.

**Figure 6:**
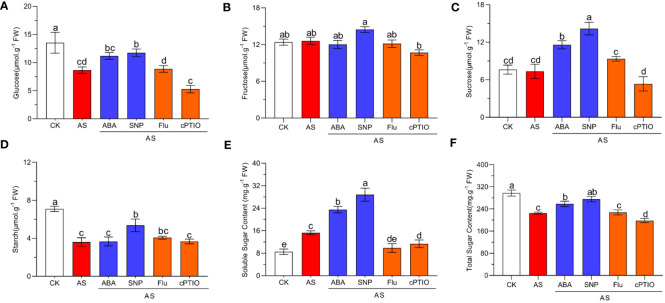
Effects of different treatments on sugar accumulation in *N. tangutorum* seedlings. 40-day-old seedlings were treated with different treatments (100 mM alkali, 100 mM alkali +15 μm ABA/5μm Flu, 100 mM alkali +50 μm SNP/70 μm cPTIO) for 12 days(AS: NaHCO_3_: Na_2_CO_3 =_ 9:1, pH=9.5). Plants grew in Hoagland solution served as controls (CK). **(A–F)**: Changes in the accumulation of carbohydrates in leaves of *N. tangutorum* seedlings under different stress treatments, in order: glucose, fructose, sucrose, starch, soluble sugar and total sugar. Bars represent the means ± SDs of three replicates. Significant differences among treatments are indicated by different letters within a panel based on Duncan’s multiple range test (*P* < 0.05).

Alkali stress seriously affects the photosynthesis of plants, which may lead to changes in carbohydrate products of photosynthesis. We detected the changes in related carbohydrate substances under different treatments. The results showed that alkali stress significantly reduced the contents of glucose, starch, and total sugar but had no significant effect on fructose and sucrose. Compared with single alkali stress, combined ABA and SNP treatment significantly increased the contents of glucose, starch, and total sugar, especially sucrose content significantly increased by two times but did not affect fructose content (**Figures 6A–F**). In addition, alkali stress significantly induced soluble sugar accumulation compared with normal conditions, which increased by 1.78 times compared with control. External application of ABA and SNP increased soluble sugar content to 2.73 and 3.34 times (**Figure 6E**). In general, SNP application was better than ABA in promoting the accumulation of sugars under alkali stress, especially for sucrose, starch, and soluble sugars (**Figure 6**). The application of Fluorine and cPTIO has the opposite effect. In conclusion, the combined application of ABA and SNP effectively alleviated the photosynthetic inhibition caused by alkali stress and promoted the accumulation of photosynthate sugars, and the promotion effect of SNP was better than ABA.

### 3.7 ABA and SNP promoted the accumulation of flavonoids in *N. tangutorum* seedlings under alkali stress

**Figure 7:**
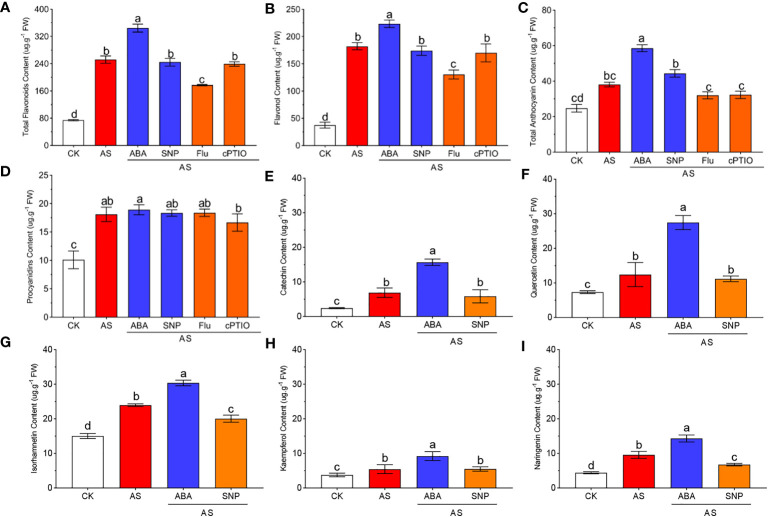
ABA and SNP promote the accumulation of flavonoid and anthocyanin secondary metabolites under alkaline stress. 40-day-old seedlings were treated with different treatments (100 mM alkali, 100 mM alkali +15 μm ABA/5 μm Flu, 100 mM alkali +50 μm SNP/70 μm cPTIO) for 12 days (AS : NaHCO_3_: Na_2_CO_3 =_ 9:1, pH=9.5). Plants grew in Hoagland solution served as controls(CK). **(A)**: Total flavonoid content; **(B)**: Flavanol content; **(C)**: Total anthocyanin content; **(D)**: Proanthocyanidin content; **(E)**: Catechin content; **(F)**: Quercetin content; **(G)**: Isoamnetin rhcontent; **(H)**: Kaempferol content; **(I)**: Naringenin content. Bars represent the means ± SDs of three replicates. Significant differences among treatments are indicated by different letters within a panel based on Duncan’s multiple range test (*P* < 0.05).

Flavonoids are a class of powerful antioxidants widely recognized to play a role in plant resistance to adversity. To evaluate the role of secondary metabolites of flavonoids in response to alkali stress in *N. tangutorum* seedlings and the regulatory role of ABA and SNP in the accumulation of flavonoids, we examined the content of flavonoids under different treatments and the expression levels of key genes in the metabolic pathway. We found that alkali stress significantly induced the accumulation of total flavonoids, total anthocyanins and flavonols. Combined application of ABA further promoted the accumulation of these compounds, but SNP had no significant effect (**Figures 7A–C**). Compared with CK, proanthocyanidins accumulated obviously under alkali stress, but the external application of ABA and SNP had no further promoting effect on the proanthocyanidins (**Figure 7D**). The application of Fluorine and cPTIO has the opposite effect. The contents of several flavonoids were further detected, and the results were consistent with the above results. Alkali stress significantly induced the accumulation of flavonoids such as naringin, quercetin, isorhamnetin, kaempferol, and catechin, which increased significantly to more than 2 times CK, and among them, catechin even reached three times CK. Exogenous application of ABA could further promote the induction effect, and the contents of several flavonoids were further increased to 3.25 times, 3.74 times, 2.01 times, 2.44 times and 6.47 times of CK, among which the content of isorhamnetin was the highest, and the increase of catechin was the largest. However, SNP also had little effect on the accumulation of several flavonoids (**Figures 7E–I**).

**Figure 8:**
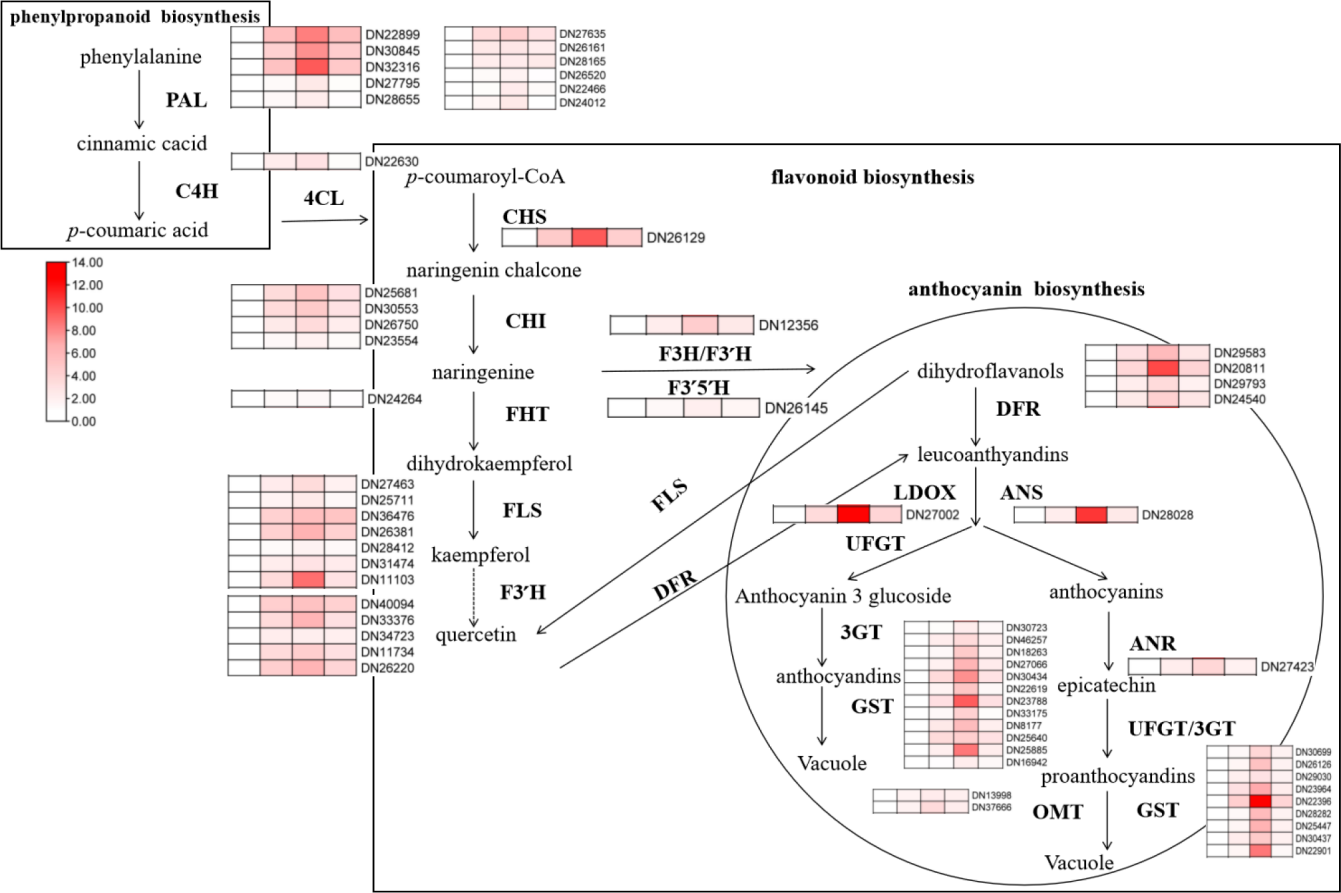
RT-PCR analysis of the effects of exogenous ABA and SNP on the expression of related genes in the synthesis pathways of phenylpropane, flavonoids and anthocyanins under alkaline stress. 40-day-old seedlings were treated with different treatments (100 mM alkali, 100 mM alkali +15 μm ABA, 100 mM alkali +50 μm SNP) for 12 days (AS : NaHCO3: Na_2_CO_3 =_ 9:1, pH=9.5). Plants grew in Hoagland solution served as controls (CK). The detected genes include: *NtPAL*(DN22899/DN30845/DN32316/DN27795/DN28855),*NtC4H*(DN22630),Nt4CL(DN27635/DN26161/DN26165/DN26520/DN22466/DN24012), *NtCHS*(DN26129), *NtCHI*(DN25681/DN30553/DN26750/DN23554), *NtFHT*(DN24264), *NtFLS*(DN27463/DN25711/DN36475/DN26381/DN28412/DN31474/DN11103), *NtF3’H* (DN40094/DN33376/DN34723/DN11734/DN26220), *NtF3H*(DN12356), *NtF3’5’H* (DN26145), *NtDFR*(DN29583/DN20811/DN29793/DN2454 0), *NtLDOX*(DN27002), *NtANS*(DN28028), *NtANR*(DN27423), *NtUFGT*(DN30699/DN26126/DN29030/DN23964/), *Nt3/5/7GT*(DN22396/DN28282/DN25447/DN30437/DN22901),*NtGST*(DN30723/DN46257/DN18263/DN27066/DN30434/DN22619/DN23788/DN33175/DN8177/DN25640/DN25885/DN16942),*NtOMT*(DN 13998/DN37666).

At the same time, we detected the expression levels of key genes in the pathway of flavonoid synthesis under different treatments, including five phenylalanine lyases (PALs), one cinnamate hydroxylase (C4H), six coumadany-CoA ligases (4CLs), one Chalcone synthase (CHS), four Chalcone isomerases (CHI), one Fatty ω-hydroxy acid/fatty alcohol hydroxycinnamoyl transferase (FHT), seven flavonol synthases (FLS), one flavanone-3-hydroxylase (F3H), five flavonoid-3 ‘-hydroxylases (F3’H), one flavonoid-3’5’-hydroxylase (F3’5’H), four dihydroflavonol reductases (DFR), two anthocyanin synthases (ANS/ANR), one colorless anthocyanin dioxygenase (LDOX), four flavonoidyltransferases (UFGT), five flavonoid 3/5/7-O-glycosyltransferases (3GT/5GT/7GT), twelve glutathione S-transferases (GST), and two O-methyltransferases (OMT). The results showed that alkali stress significantly induced the expression of genes related to the synthesis of flavonoids, and the combined application of ABA further significantly promoted the transcription of these genes. Compared to the controls, alkali stress increased *NtCHS/NtFLS/NtF3’H/NtF3H/NtANR/NtDFR/NtUFGT/NtGST* expression 3–4 times. Combined application of ABA further promoted the expression of the above genes, and the expression levels of *NtDFR/NtUFGT/NtGST* increased to 4–5 times that of alkali stress, indicating that exogenous application of ABA can significantly promote the transcription level of flavonoids synthesis genes under alkali stress. However, the expression of the above genes was almost consistent with the single alkali stress when SNP was applied, which was consistent with its content change (**Figure 8**).

Under alkaline salt stress, *N. tangutorum* seedlings can reduce oxidative damage and osmotic stress by regulating the expression level of genes related to the accumulation of flavonoids’ secondary metabolites and accumulating anthocyanins and flavonols in large quantities. The external application of ABA had an important regulatory effect on the accumulation of flavonoids and anthocyanins, while SNP was no involved in the relevant regulation.

## 4 Discussion

Plants have developed complex regulatory mechanisms to cope with environmental stress and accumulate nutrients during long-term evolution. Some gas signaling molecules (e.g., NO and CO) and hormones (e.g., ABA, ethylene, and Brassinosteroids) have played important roles in this process. Similar to ROS such as H_2_O_2_, NO, and its derived molecules (NO_2_, ONOO^−^, GSNO) are collectively referred to as active nitrogen (RNS), which act as signaling molecules to regulate various physiological processes in plants (Fancy et al., 2017). ABA is considered one of the most important anti-stress hormones in plants, regulating the expression of multiple stress response genes, inducing SnRK family protein kinase activity and MAPK signaling cascade defense response (Huang et al., 2017). During a plant stress response, NO plays an important role in regulating the production of reactive oxygen species (ROS) induced by ABA in regulating the activities of plant antioxidant systems such as catalase (CATs), superoxide dismutase (SODs) and ascorbic acid-glutathione cycle. (Garcia-Mata and Lamattina, 2007). For example, in tall fescue seedlings, NO participated in ABA-mediated plant tolerance response to low light stress by regulating the antioxidant defense system and high photosynthetic rate.

However, there are few studies on the regulation of ABA and SNP in response to alkaline stress at high pH (Zhang et al., 2018). This study found that 100 mM alkaline salt (pH= 9.5) treatment significantly inhibited the growth of *N. tangutorum* seedlings, resulting in decreased photosynthesis, oxidative damage, osmotic regulation imbalance, and ion toxicity. The combined application of 15 μM ABA and 50 μM SNP could partially restore the photosynthetic efficiency and ion homeostasis of *N. tangutorum* seedlings, further improve the reactive oxygen scavenging capacity of the antioxidant system and the accumulation of flavonoids secondary metabolites, and alleviate the growth inhibition and physiological damage caused by alkali stress (**Figure S1** and **Table 1**). Among them, SNP significantly improved the photosynthetic efficiency and the regulation of carbohydrate accumulation more than ABA; ABA had a more positive effect on regulating the accumulation of flavonoids’ secondary metabolites and improving the efficiency of the antioxidant system. Our results suggest that ABA and SNP play a positive role in alleviating the adverse effects of alkali stress on *N. tangutorum* seedlings from different angles.

### 4.1 Exogenous ABA and SNP increase ABA and NO content in plants under alkali stress, reduce oxidative damage and Na^+^ toxicity

Under environmental stress, plants activate complex signal transmission networks, causing physiological changes in response to adverse external conditions. Oxidative damage is a major harm to plants caused by alkali stress, severely restricting plant growth and development and reducing plant biomass (Klay et al., 2018; Sun et al., 2019). In this study, alkali stress of 30–100 mM resulted in significant increases in the contents of H_2_O_2_, O_2_-, MDA, and EL, which caused serious oxidative damage to *N. tangutorum* seedlings and significantly reduced the growth indicators, such as plant height, fresh weight(FW), relative water content(RWC), and degree of succulent(DS) (**Figure 1**). It has been found that multiple environmental stresses stimulate the generation of endogenous ABA and NO in plants, such as drought and alkali stress that induce significant ABA accumulation in wheat and maize, respectively (Ma et al., 2018; Wang et al., 2022), which promoted their adaptability to the adverse environment through regulating photosynthesis, antioxidant pathways, ion balance and other pathways. It has been reported that exogenous application of ABA or SNP can effectively promote the production of ABA or NO in plants and reduce the damage caused by stress, such as *Brassica pekinensis* (Ren et al., 2020), *Sorghum bicolor* (Sun et al., 2019), and *Medicago sativa* (Li, X et al., 2020). In this study, alkali stress also induced a significant accumulation of ABA and NO, and the expression of ABA and NO synthesis and signal transduction-related genes, suggesting that ABA and NO may be involved in the regulation of *N. tangutorum* seedlings’ response to alkali stress (**Figure S2**). Exogenous application of 15 μM ABA and 50 μM SNP under alkali stress further promoted the accumulation of ABA and NO in plants, effectively alleviated the burst of ROS caused by stress, and reduced the oxidative damage and growth inhibition. The H_2_O_2_, O_2_^-^, MDA, and EL levels were significantly reduced.

Oxidative stress is plants’ main problem when they encounter harsh environments. The stronger the scavenging ability of the antioxidant system to the excess accumulation of ROS, the smaller the oxidative damage caused to it. Therefore, plants activate antioxidant systems through various regulatory pathways(Gupta et al., 2020), including ABA and NO signaling pathways. For example, exogenous SNP can increase SOD activity in cucumber seedlings, thus promoting the transformation of H_2_O_2_ into H_2_O and O_2_; meanwhile, it can enhance the activity of H_2_O_2_ scavenging enzymes (CAT, APX and GPX) and reduce the accumulation of ROS in root cell mitochondria, thus alleviating oxidative damage caused by salt stress (Shi et al., 2007). Exogenous ABA can significantly improve the activities of antioxidant enzymes, including POD, SOD, CAT, and APX, and promote the ability of tall fescue to clear newly generated ROS, thus reducing oxidative damage (Zhang et al., 2018). In this study, the ROS outbreak occurred in *N. tangutorum* seedlings under alkali stress, and the antioxidant system of the plants was also significantly induced, with the activity of antioxidant enzymes/GR/GST/CAT/APX/SOD and the content of antioxidant GSH significantly increased. After exogenous application of 15 μM ABA and 50 μM SNP, the above antioxidant enzyme activity and antioxidant content were further enhanced, which significantly inhibited the excess accumulation of ROS and oxidative damage, thus improving the physiological activity of plants from many aspects and alleviating the damage caused by alkali stress to plant growth. Interestingly, external ABA administration had a more significant positive effect than SNP (**Figure 2**).

Saline-alkali stress usually keeps a high Na^+^ and Na^+^/K^+^ ratio in plant cytoplasm, leading to Na^+^ toxicity. High Na^+^ and pH values in the external environment under alkali stress can lead to the increase of Na^+^ and H^+^ concentration gradient inside and outside the cell, resulting in excessive accumulation of Na^+^ in the cell and affecting the growth and development of plants. For example, high pH will destroy the inhibiting ability of wheat seedlings to absorb Na^+^, resulting in excessive accumulation of Na^+^ induced by alkali stress(Li et al., 2020). Under high pH stress, Na^+^ content in the leaf and stem of *Hippophae rhamnoides* L. accumulated significantly, resulting in significant ion toxicity (Chen et al., 2009). Studies have shown that the exogenous application of ABA and SNP can regulate the pattern of ion accumulation in plants and maintain ion homeostasis (Chen et al., 2013; Chen et al., 2020). For example, SNP pretreatment can significantly reduce the content of Na^+^ and increase the content of K^+^ in the radicle and germ of *Brassica napus* under salt stress, thus reducing the ratio of Na^+^ to K^+^(Hasanuzzaman et al., 2018). In rice under salt stress, exogenous application of ABA regulates sodium and potassium ion homeostasis by promoting Na^+^ outflow and K^+^ inflow, thus enhancing the tolerance to saline-alkali stress (Li et al., 2020). In this study, alkali stress significantly reduced the K^+^ content in the roots of *N. tangutorum* seedlings and significantly increased the Na^+^ and Na^+^/K^+^ in the shoots and roots. From the results of quantitative gene analysis, the expression of plasma membrane-located Na^+^/H^+^ reverse transporter (*NtSOS1*) and vacuole-located Na^+^/H^+^ reverse transporter (*NtNHX1/2/3*) in leaves and roots was significantly up-regulated, while the transcription level of plasma membrane-located high-affinity K^+^ transporter (*NtHKT1*) was significantly down-regulated. The expression level of K^+^/H^+^ reverse transporter 5 (*NtKEA5*) located in the Golgi apparatus reverse mask capsule was significantly increased in the shoots (**Figures 3D, E**). The *N. tangutorum* seedlings mobilized the ion transport system to transport Na^+^/K^+^ ions to a certain extent, but it still could not effectively prevent the excessive accumulation of Na^+^ and the blocked absorption of K^+^ in the shoots and roots, resulting in obvious ion toxicity and growth inhibition. When 15 μM ABA and 50 μM SNP were applied under alkali stress, the accumulation of K^+^ in the roots of *N. tangutorum* seedlings was effectively promoted, and the content of Na^+^ and the ratio of Na^+^/K^+^ in the shoots and roots were significantly reduced, which played a positive role in maintaining the homeostasis of Na^+^/K^+^ ions in plants.ABA and SNP significantly induced the transcription of the Na^+^ transporter gene (*NtSOS1/NtNHX1/3*) in the shoots and roots, and the K^+^ influx transporter gene (*NtHAK3/6/12/KUP4/KEA3*) in the root (**Figures 3A–D**). The synergistic effect of these ion transporters made the outflow of Na^+^ in root and abovemonial cells greater than the inflow, which significantly reduced the content of Na^+^ in cells compared with the single alkali stress. At the same time, the content of K^+^ in the root was significantly increased, effectively reducing the Na^+^/K^+^ imbalance caused by alkali stress.

In conclusion, ABA and SNP further regulate the ion accumulation pattern in plants by regulating the expression of Na^+^ and K^+^ transporter-related genes, thus alleviating the ion toxicity caused by alkali stress on *N. tangutorum* seedlings.

### 4.2 Stomatal movement and water loss in leaves of *N. tangutorum* seedlings under alkali stress regulated by ABA and SNP

In response to adverse environments, plants usually take a series of self-protection measures, such as regulating stomatal movement and reducing transpiration (Wang, Z et al., 2020). The regulation of stomatal opening is not only regulated by plant genetic genes but also influenced by various external environmental factors such as atmospheric CO_2_ concentration (Gray et al., 2000), heavy metals (Gonçalves et al., 2020), salt damage (Hu et al., 2013), light(Wei et al., 2020), hormones, and other signaling molecules (Huang et al., 2022). Studies have shown that the external application of ABA and SNP can induce stomatal closure and reduce stomatal density and conductance (Li et al., 2000; García-Mata and Lamattina, 2002). Salt and alkali stress can lead to the irregular spatial distribution of stomata in plants, decrease stomatal density, openness and conductance (Pompelli et al., 2021), reduce CO_2_ concentration in leaves, and ultimately lead to a decrease in net photosynthetic rate (Shabala et al., 2012). In this study, the leaf stomatal width aperture of *N. tangutorum* seedlings decreased significantly under alkali stress. In contrast, leaf surface temperature and water content decreased significantly. Water loss increased (**Figure 4**). Under alkali stress, applying ABA and SNP further reduced stomatal opening and water loss and recovered the decrease of leaf surface temperature and water content caused by transpiration, which effectively alleviated the water loss and physiological inhibition caused by alkali stress. The maintenance of the water retention capacity of plants is also closely related to the accumulation of osmotic regulatory substances. Studies have shown that the external application of ABA and SNP can effectively activate the osmotic protection system of plants and improve the water retention capacity of plants. For example, under salt stress, ABA can promote the accumulation of osmoprotective substances, such as proline in plants, and prevent the separation of cytoplasmic walls due to water loss in cells (Hewage et al., 2020). Exogenous SNP effectively improved the activity of antioxidant enzymes and the content of osmotic regulatory substances in pepper seedlings under salt stress to alleviating the inhibition caused by salt stress (Shams et al., 2019). In this study, the exogenous addition of 15 μM ABA and 50 μM SNP significantly induced the accumulation of osmotic regulating substances, such as betaine, soluble protein and proline in plants under alkali stress, which played a positive role in maintaining the osmotic pressure stability of plants and improving the water retention ability of plants. When SNP was applied externally, the accumulation of osmoregulatory substances in plants and water content in leaves were significantly higher than that of exogenous addition of ABA, indicating that NO had a better regulating effect on osmotic balance and reduced water loss in *N. tangutorum* seedlings under alkali stress (**Figure 4**).

### 4.3 Exogenous ABA and SNP regulate photosynthetic efficiency and accumulation of photosynthate in *N. tangutorum* seedlings under alkali stress

Photosynthesis provides material and energy for plant growth and is an important life process for plants. The factors leading to decreased plant photosynthesis can be divided into stomatal and non-stomatal restrictions (Tsai et al., 2019). Under environmental stress, the decrease of stomatal conductance leads to the decrease of CO_2_ entering stomata and the failure to meet the requirements of normal photosynthesis, which is called the stomatal restriction of photosynthesis. Non-stomatal limiting factors mainly refer to the destruction of chloroplast structure, the decrease of photosynthetic pigment content and photosynthase activity, and the destruction of reactive oxygen metabolism (Wu et al., 2014). Studies have found that environmental stress decreases chlorophyll content and plants’ photosynthetic rate (Hazrati et al., 2016). In this experiment, it was found that the stomata of *N. tangutorum* seedlings were partially closed under alkali stress, resulting in a significant increase in intercellular CO_2_ concentration compared with CK. At the same time, chlorophyll content, Pn rate and other non-stomatal limiting factors were significantly decreased, which indicated that non-stomatal limiting factors might be the main factors affecting the photosynthesis of *N. tangutorum* seedlings. 100 mmol/L alkali stress significantly reduced Fv/Fm in the leaves, indicating that the PSII reaction center was damaged by photoinhibition and the photosynthetic activity decreased (**Figure 5**). This is consistent with the conclusion of Conceicao Vieira Santos.etal in sunflower, indicated that the decrease of φPSII and qP further confirmed the photosynthetic electron transfer was inhibited, PSII light energy conversion efficiency was reduced, and excess excitation energy was increased (Scartazza et al., 2020). NPQ decreased to the lowest level under alkali stress, indicating that excess excitation energy may cause further damage to the chloroplast structure. Studies in cucumber and maize have shown that the addition of exogenous ABA and SNP can alleviate the decrease of chlorophyll content under salt stress, enhance the absorption and utilization of light energy by chloroplasts, promote the assembly of thylakoid membrane pigment-protein complex under iron deficiency conditions, and improve the photosynthetic rate (Hao et al., 2021; Rehaman et al., 2022). Many studies have shown that SNP can not only directly remove reactive oxygen species but also activate the activity of the antioxidant system (Corpas et al., 2019), protect the integrity of chloroplast structure and function, and improve photosynthetic efficiency (Pucciariello and Perata, 2017). In this study, exogenous application of 15 μm ABA and 50 μm SNP could effectively alleviate the decrease of chlorophyll content caused by alkali stress and significantly increase the Fv/Fm, qP, φPSII, and NPQ. These results indicate that exogenous ABA and NO could reduce the damage to chloroplast structure and function caused by oxidative damage by improving the scavenging capacity of ROS in the antioxidant system, preventing chlorophyll degradation, and partially restoring the photosynthetic efficiency of *N. tangutorum* seedings, thus alleviating the inhibition of plant photosynthesis in alkali stress. Meanwhile, the recovery of photosynthetic efficiency may be achieved by increasing the utilization rate of light energy rather than the heat dissipation of excitation energy. In addition, our physiological indicators showed that SNP had a more significant effect on the restoration of the photosynthetic system than ABA (**Figure 5**). However, the involvement of ABA and SNP in signal transduction and their molecular mechanisms in photosynthetic regulation under alkali stress remain to be further explored.

As autotrophs, plants can assimilate inorganic carbon into sugars through photosynthesis to meet their own energy requirements (Hennion et al., 2019). Under a given abiotic stress condition, sugar plays the role of osmoprotectant and is part of the reactive oxygen scavenging system (Hennion et al., 2019; Salmon et al., 2020). In this study, the accumulation of photosynthates under various treatment conditions was detected, and it was found that alkali stress significantly reduced the contents of glucose, starch and total sugar but had no significant effect on fructose and glucose. But soluble sugar content was significantly up-regulated. This may be because *N. tangutorum* is a resistant plant, and soluble sugar plays an important role in osmotic regulation (Salmon et al., 2020). The contents of glucose, fructose, starch, soluble sugar and total sugar were significantly increased after ABA and SNP were applied under alkali stress. This may be because ABA and NO can improve the photosynthetic efficiency of plants, stimulate the metabolism of carbohydrates, and promote the accumulation of photosynthates under alkali stress. Similarly, the promotion effect of SNP on carbohydrate accumulation was significantly better than ABA, which was consistent with the alleviating ability of SNP and ABA on photosynthetic inhibition under alkali stress (**Figure 6**).

### 4.4 Exogenous ABA and SNP promoted the accumulation of flavonoids in *N. tangutorum* seedlings under alkali stress

Flavonoids are common secondary metabolites, including flavonols, anthocyanins, and others. It has many biological functions, including defense against biological and abiotic stresses. As important secondary metabolites in plants, flavonoids play an important role in pollen fertility (Wang, L et al., 2020), polar auxin transport (Zhang et al., 2021), and plant resistance to stress injury (Shomali et al., 2022). Previous reports have shown that the accumulation of flavonoids can remove stress-reactive elements from cells, such as free radicals, monopolistic oxygen molecules and peroxides (Wang et al., 2016)and reduce their levels after ROS formation, thus exerting antioxidant functions and enhancing plant tolerance to abiotic and biological stresses (Agati et al., 2012). The study found that under salt stress, drought stress, and MEJA treatment, the content of flavonoids in plants increased significantly to improve their tolerance to abiotic stress (Li, Y et al., 2020; Ahmed et al., 2021). In our study, alkali stress caused a large amount of flavonoid accumulation in *N. tangutorum* seedlings, indicating that flavonoids played a positive role in the removal of excessive ROS and osmotic regulation of *N. tangutorum* seedings. Exogenous application of ABA under alkali stress further increased the accumulation levels of total flavonoids, flavonol, and total anthocyanins, indicating that the ABA signaling pathway plays an important regulatory role in the accumulation of flavonoids, while SNP has little effect on the accumulation of flavonoids (**Figure 7**).

Phenylalanine ammonia-lyase (PAL) is a synthase in the phenylpropane-like metabolic pathway, which can catalyze the deamination of L-phenylalanine (L-Phe) to trans-cinnamic acid and is the direct or indirect precursor of many secondary metabolites (Jun et al., 2018). Flavonoids are also synthesized through the phenylpropanoid pathway. Phenylalanine acts as the precursor molecule for flavonoid biosynthesis, which is transformed to cinnamic acid by phenylalanine ammonia lyase (PAL) (Anwar et al., 2019). A large number of studies have shown that PAL activity is significantly positively correlated with the accumulation of lignin, anthocyanins, and flavonoids play an important role in regulating the generation of secondary metabolites, maintaining normal plant growth and development, and improving plant resilience (Anwar et al., 2021; Peng et al., 2022). It was found that the wild-type *Arabidopsis thaliana* could accumulate anthocyanins to resist the stress under UV-B stress, but the double mutant pal1-pal4 could not synthesize anthocyanins normally, resulting in greater growth inhibition of the stress than the wild-type (Huang et al., 2010).In this study, alkali stress significantly up-regulated the PAL gene of phenylpropane, and SNP application did not increase its expression level and accumulation of flavonoids. After the exogenous application of ABA, the expression of PAL-related genes was significantly up-regulated, and the accumulation of flavonoids in the phenylpropane metabolic pathway was more significant (**Figures 7A–D**). The accumulation of these substances played a crucial role in the response of *N. tangutorum* seedlings to alkali stress. Flavonols and anthocyanins are the most important and abundant flavonoids, which have antioxidant properties in plants(Martens et al., 2010; An et al., 2016). As a key enzyme in Flavonol synthesis, Flavonol synthase (FLS) can control flavonol entry into the branch pathway of flavonol synthesis and form various flavonol compounds, mainly in the form of quercetin and kaempferol(Falcone et al., 2012). Some studies have found that exogenous ABA can significantly induce the accumulation of plant flavonoids under stress. For example, exogenous ABA can significantly induce the accumulation of flavonols such as kaverol in tea leaves and the high expression of genes related to flavonol synthesis, such as *CHI*, *DFR*, *F3’H* and *FLS* under drought stress (Gai et al., 2020). Exogenous application of ABA-induced large accumulation of *CHS* and flavonoid metabolites, which were key enzymes in flavonoid synthesis, and improved drought tolerance of *pigeon pea* (Yang et al., 2021). In this study, we found that external application of ABA significantly increased the accumulation of flavonoids such as naringin, quercetin, isorhamnetin, kaempferol and catechin in *N. tangutorum* seedlings under alkali stress, among which the increases of flavonols such as quercetin, kaempferol, and isorhamnetin were the most significant (**Figures 7E-I**). At the same time, the transcription levels of *CHS*, *CHI*, *FLS* and other enzyme synthesis genes that regulate the synthesis of these flavonols were also significantly up-regulated compared with alkali stress alone (**Figure 8**). Anthocyanins are a kind of water-soluble natural pigments belonging to flavonoids, which are widely found in angiosperms and are important components formed in the process of plant growth. Anthocyanins play an important role in improving the ability of plants to withstand stress and stress and are of great significance for plant growth, reproduction and environmental adaptation (Fan et al., 2016; Liang and He, 2018).

The structural genes of the anthocyanin synthesis pathway can be divided into prophase biosynthetic genes (e.g., CHS, CHI, and F3H) and late biosynthetic genes (e.g., DFR, ANS and UF3GT). It was found that *PAL*, *C4H*, *4CL*, *CHS*, *CHI*, *F3H*, *DFR*, *LDOX*, and *UFGT* are key structural genes involved in anthocyanin synthesis and are important enzyme reactions involved in the anthocyanin synthesis pathway (Zhang and Schrader, 2017). The increased expression of these genes can lead to the activation of the anthocyanin synthesis pathway of *Arabidopsis thaliana*, thus increasing the anthocyanin content in plants (Petroni and Tonelli, 2011). Some genes can regulate these anthocyanin synthesis genes, such as *MYB*. Overexpression *NtMYB2* down-regulates both early and late key anthocyanin biosynthesis pathway genes. In addition, *NtMYB2* down-regulated the proanthocyanidin (PA) biosynthetic pathway gene *NtANR* in tobacco flowers (Anwar et al., 2018). This study found that total anthocyanins and proanthocyanidins were significantly accumulated in *N. tangutorum* seedlings under alkali stress (**Figures 7C, D**), and their expression of their related synthetic genes were up-regulated (**Figure 8**). After exogenous application of ABA, the total anthocyanin content was significantly increased compared with that under alkali stress (**Figure 7C**), and the expressions of *PAL*, *C4H*, *4CL*, *CHS*, *CHI*, *F3H*, *DFR*, *ANS*, *LDOX* and *UFGT* were all increased to varying degrees. The accumulation of anthocyanins and the expression of related genes were not significantly affected by SNP application. As excellent antioxidants, the increased flavonoid content plays a positive role in effectively removing ROS (An et al., 2016). In this study, the exogenous application of ABA was more effective than SNP in preventing the accumulation of ROS caused by alkali stress (**Figure 2**). On the one hand, ABA is better than SNP in inducing the increase of antioxidant enzyme activity in plants (**Figure 2**). On the other hand, exogenous application of ABA can significantly promote the accumulation of secondary metabolites of flavonoids under alkali stress than SNP and further improve the scavenging capacity of ROS in the antioxidant system, thus alleviating the physiological damage and growth inhibition of plants (**Figures 2**, **7**).

## 5 Conclusions

In conclusion, *N. tangutorum* seedlings had strong alkaline resistance and could tolerate 100 mM alkali stress (NaHCO_3_:Na_2_CO_3 =_ 9:1) for 12 days. The accumulation of flavonoids and anthocyanins was higher during the period. Exogenous application of ABA and SNP could significantly reduce oxidative damage and Na^+^ ionic toxicity, and alleviate the growth inhibition of *N. tangutorum* seedlings under alkali stress. They can also improve photosynthetic efficiency and promote the accumulation of flavonoids. Compared with ABA, SNP significantly promoted the accumulation of chlorophyll under alkali stress, increased the photosynthetic indexes such as Pn, Fv/Fm, φPSII and ETRII, thus improved the photosynthesis and accelerated the accumulation of glucose/fructose/sucrose/starch and total sugar. Compared with SNP, ABA can significantly promote the contents of total flavonoids, total anthocyanins and flavonols, improve the transcription level of the synthesis pathway of flavonoid metabolites such as *NtFLS/NtF3’H/NtF3H* etc genes, and the accumulation of naringin, quercetin, isorhamnetin, kaempferol and catechin. In a word, the effects of SNP on the improvement of photosynthetic efficiency and the regulation of carbohydrate accumulation under alkali stress were significantly better than ABA, and the effects of ABA on the regulation of flavonoids and anthocyanin secondary metabolites accumulation were more significant. At the same time, both of them can play a positive role in the defense response of *N. tangutorum* seedlings to alkali stress.

## Data availability statement

The original contributions presented in the study are included in the article/**Supplementary Material**, further inquiries can be directed to the corresponding author.

## Author contributions

JZ and KC contributed to the design and performance of experiments, analyses, data interpretation, and manuscript drafting. YW contributed to study conception and design, revision, and final approval of submission. XL, ZD and LZ contributed to plant cultivation and measurement of physiological indicators. All of the authors have read and approved the final manuscript. All authors contributed to the article and approved the submitted version.
