# Supplementary material for: Exogenous abscisic acid and sodium nitroprusside regulate flavonoid biosynthesis and photosynthesis of Nitraria tangutorum Bobr in alkali stress
Source: Front Plant Sci. 2023 Mar 15;14:1118984. doi: 10.3389/fpls.2023.1118984 (PMC10057120; doi:10.3389/fpls.2023.1118984)
Supplement: Supplementary file 1 [file DataSheet_1.docx]

## Supplementary Material

## 1.Supplementary Figure

Figure S1. ABA and SNP affect the growth of *N. tangutorum* seedlings in a concentration-dependent manner. Growth status of Tanggulan seedlings under with different concentrations of ABA and SNP. Selected 40-day-old plants were treated with different concentrations of ABA(10/15/30 μM), SNP(30/50/70 μM),cPTIO(70 μM) and Fluridone (5 μM)under 100mM alkali stress for 12 days(AS:NaHCO_3_: Na_2_CO_3_=9:1, pH=9.5). Plants grown in Hoagland's solution served as positive controls. Fluridone(Flu) and cPTIO solutions served as negative controls under 100mM alkali stress.

Figure S2. Detection of ABA and NO content and related gene expressions under different treatments. 40-day-old seedlings were treated with different treatments (100 mM alkali, 100 mM alkali +15 μm ABA/5 μm Flu, 100 mM alkali +50 μm SNP/70 μm cPTIO) for 12 days (AS:NaHCO_3_: Na_2_CO_3_=9:1，pH=9.5). Plants grew in Hoagland solution served as controls (CK). (A): Expression levels of synthesis and signaling-related genes of ABA and NO ,including*NtNOA1, NtNR2, NtABF1, NtABF3, NtABI5, NtNCED1/3/4/5, NtAAO, NtSDR, NtPYL2/6, NtPP2C* and *NtSnRk2.2/2.3*; (B):NO content; (C):ABA content. Bars represent the means ± SDs of three replicates. Significant differences among treatments are indicated by different letters within a panel based on Duncan’s multiple range test (*P* < 0.05).

## 2.Supplementary Tables

**Table S1  The qRT-PCR specific primers for gene validation in *N. tangutorum***

| **Gene ID** | **forward primer(5’-3’)** | **reverse primer(5’-3’)** |
| --- | --- | --- |
| *Nt-Actin* | GGAATCCACGAGACCACCTACA | GATTGATCCTCCGATCCAGACA |
| *NtSOS1* | TGATGGAGGTTCTGCC | CCACGGAGTAAATGTCTG |
| *NtHKT1* | GAGCGACCCACTAAAC | TACTCCATCTGCCCAC |
| *NtKCO* | GCTAGCGTAGACC | AAGGTCGTGCGA |
| *NtKUP4* | CACTCATATCGGCAACA | AACCAGGGCATAGAGG |
| *NtHAK6* | CATCAGTCAGGGCA | CTGGAAGGCGCGGTA |
| *NtHAK12* | GCGTACGTGCGT | GGGTCATACAAAG |
| *NtKEA2* | GCTAGTACGCCGGT | TGCGTCGGCATC |
| *NtKEA3* | GTCTCTGCTGCGGGG | ATCGTGTCAGTGCGG |
| *NtKEA5* | ACGGAATAGTAATAATGCTC | CTTGTAGAACGCCACC |
| *NtNHX1* | ACGCCAGCAGATTCCT | CGATGATACAAACGCAGAG |
| *NtNHX2* | TAGCAAGCGGGCAAGA | CGGAGTCATCAGGAGCA |
| *NtNHX3* | CTCCCTCACATCAACTCA | CACTAACAAGCCCAACTA |
| DN22899 | GACTAGCTAGCATCGA | GGACCGTACGATCGG |
| DN30845 | ACGTACGTAGTCA | GGTACGTCAGCATG |
| DN32316 | GGTCAGTAGC | ACGTACGTACGC |
| DN27795 | ACGTAGCGTACGGTC | ACGTCAGTCAGGGG |
| DN28855 | ACGTCGTAGCGAT | CGTACAGTCAGT |
| DN22630 | GGCATCGACGT | CCGATGTGCGTA |
| DN27635 | GGCATGTGTGAC | GGTCAGTCGTCA |
| DN26161 | CAGTAGCAGTAC | GTCAGTCGACGTA |
| DN26520 | GGCCAGTACAGTA | ACGTGCACGGGTC |
| DN22466 | AGCTGCATGCAGT | CGTCACGCACTTTGG |
| DN24012 | ACGTAGCGTACGTACG | CGTCGACTACTTAA |
| DN26129 | GGTACGTTGCAGT | CCACACGTCACTT |
| DN25681 | AGCTAGCTAGCGAT | AGCCACCCGGTACCC |
| DN30553 | CGTACGTACGTAC | AACCGTCGACTAC |
| DN26750 | ACGTACGCTAGC | GGGGCCTTACACG |
| DN23554 | TTGACAGTACGTA | GGGCCCTTTTTACAC |
| DN24264 | ACGTAGCTAGCGATA | GTCACGGTCACGT |
| DN27463 | ACGTAGTCGATCAGT | GTACGTCAGGTCA |
| DN25711 | GGTCTGTCAGTAC | GACGCTAGCAAAA |
| DN36475 | ACGTACGTACGTAC | ACACACCCTTCCA |
| DN26381 | ACGCAGTCAGTA | GGGCACTCGACGGG |
| DN28412 | CCCAACGTACACG | ACTGATGCAGTACAA |
| DN31474 | TTGGCCACTGGGGG | GGGCATGCATGCAG |
| DN11103 | ACTTTTCAGAT | GCTTTGCACTGCGGAA |
| DN40094 | GCAGACTAGCAT | ACTGATACGTACGAAC |
| DN33376 | ACGTGCCAGTCA | TGCGTGCACTGC |
| DN34723 | TTAGCGCTAGCA | GGGAAACCTTCAA |
| DN11734 | ACGTACGTACGA | GGGCATCGTCAGTC |
| DN26220 | AACCGCTAGCGAAAG | GACGTAGCTAGC |
| DN12356 | GACTCATGACGGGA | GGGTTTCACGTC |
| DN26145 | ACAGCTGCAGCAAC | AAATTCCCGAC |
| DN29583 | GGGACCAGTGCAG | GGGTCGTCAGATCTAGCG |
| DN20811 | TTTACAGTACGAAC | TAGCATGCGTGAGTC |
| DN29793 | GGTACGTCACAGT | GGTACGTACGTA |
| DN24540 | ACGTCGACTGCAGG | GGGTCGTACGTCC |
| DN27002 | GGGCCCACTTTCA | AATGCTGATCAG |
| DN28028 | AAACCATCACGG | GGTACGTACTGAC |
| DN27423 | GCATCAGTACAGTC | GTCAGTACTAGCGT |
| DN30699 | AAACCCGGTTCAC | ACGTAGTACGTA |
| DN26126 | GGGGCCCTACTCA | CGTCAAACGTCA |
| DN29030 | CCCCGTCACACAT | AGTCGTACGTCGTA |
| DN23964 | GGGCACACTGCA | GGGGCCCACTACG |
| DN22396 | GGTGTCACTGCA | TGCGTGCACCAAACT |
| DN28282 | GTCACGTCACGCA | GGTACGTAGGGCTA |
| DN28282 | GGGTTCACGTCA | AAACTGCTAGCGAA |
| DN25447 | GGGACACTGCA | AACGTCAGTCAA |
| DN30437 | GGGGGTCACACTGC | GGGTACGTTTAGCT |
| DN22901 | TTTGCACTTTCA | GGGACTAGGGCAA |
| DN30723 | GGGTCACACTTTAC | AATCTAGCTAGGGCA |
| DN46257 | GTCACACTCAGTTT | GGATCAATCAGTC |
| DN18263 | ACACAAATTACGC | TTTCCCACAGCAGC |
| DN27066 | AAATGCACTGCAAA | TGGGGTACGTAGGCA |
| DN30434 | GGGGCCCTGCAC | AAACGTCTCCCG |
| DN22619 | TGACGTGCACCACC | AAGCTCGACTGG |
| DN23788 | TGCACACTGCACCC | GGGTCGTCACGTCC |
| DN33175 | GGGGCCCCTTTGCG | AAATGGTGTCGTC |
| DN8177 | TTTTAAAACGCGTCA | ACTGCGTCACGTCAA |
| DN25640 | TGGGGCTCGCACG | GGGTCAAAACCCTT |
| DN25885 | GACACTGCATTTA | GGACAAAACTTGCA |
| DN16942 | AAAAACCCTTGCGGG | GGGGGCCCACTCTC |
| DN13998 | GGGTCGCGTCACAT | TTGCGCGGGACTC |
| DN37666 | CGCGGGCTAGCTAGTA | GGCTGACGTAGCGAT |
| *NtNOA1* | GGTCGATAGTACGTAG | TGACGTAGCGATCGT |
| *NtNR2* | GCTTGCTATCCCT | CCAACTGCCCAAA |
| *NtABF1* | GGAGCCAAAGACAAG | CGGTCGTTGCGTA |
| *NtABF3* | GGCGCGGGTACGT | CGTCGTCGTACG |
| *NtABI5* | CACATCTTCGGTTTCCC | TTTGCTACTTCTGCCTCC |
| *NtNCED1* | CCGTACGACGT | CGGGGTACATG |
| *NtNCED3* | GGGATAGTAGTCCGATG | TGCAGTCGATACGGGG |
| *NtNCED4* | CAACGGAGTGCCAAGTCA | CGTCAAAGAGGTGGTAAGGT |
| *NtNCED5* | AGGATTATTCGGTCTGGTT | AGAAGACGGTTGTTGAAGTAG |
| *NtAAO* | CGGTGCGTGCGATTA | CGCCTCTTTGAG |
| *NtSDR* | ACGTCGTAGCTACCGTA | GGTACAGTCTTTTGCA |
| *NtPYL2* | TATTCCCACCTCAAACCC | TCGTCGTCCAGCATCTC |
| *NtPYL6* | GAAACACTGCCATTACCAC | TCCAACGAAACTAACCATC |
| *NtPP2C* | GCGACGTAGTCAGGG | ATGCGTGCAGGTACG |
| *NtSnRK2.2* | CCGAGGAGCCAAGGAAC | GGCGGCACTCTGGAGAT |
| *NtSnRK2.3* | GCGGATGCTAGCGGTA | CGGACTGATTGCTA |
